# Costs associated with delivering HPV vaccination in the context of the first year demonstration programme in southern Mozambique

**DOI:** 10.1186/s12889-019-7338-4

**Published:** 2019-08-01

**Authors:** Sergi Alonso, Olga Cambaco, Yolanda Maússe, Graça Matsinhe, Eusébio Macete, Clara Menéndez, Elisa Sicuri, Esperança Sevene, Khátia Munguambe

**Affiliations:** 1 CISM (Centro de Investigação em Saúde de Manhiça), Bairro Cambeve, Rua 12, Distrito da Manhiça, Maputo, CP 1929 Mozambique; 2 ISGlobal, Hospital Clínic, Universitat de Barcelona, Barcelona, Spain; 3 Centre for Primary Care and Public Health, Barts and The London School of Medicine & Dentistry, Queen Mary University of London, London, UK; 4 Expanded Program on Immunisation (EPI), Ministry of Health, Maputo, Mozambique; 5 Health Economics group, School of Public Health, Department of Infectious Disease Epidemiology, Imperial College London, London, UK; 6 Faculty of Medicine, Eduardo Mondlane University, Maputo, Mozambique

**Keywords:** Human papillomavirus, HPV-vaccination, Cost estimation, Mozambique

## Abstract

**Background:**

In Mozambique cervical cancer is a public health threat, due to its high incidence and limited access to early diagnosis of precancerous lesions. International organisations are supporting the introduction of human papillomavirus (HPV) vaccines in low- and middle-income countries. Some of these countries recently conducted demonstration programmes, which included evaluation of acceptability, coverage, and practicality of implementation and of integration in existing programmes. Information on costs of delivering the vaccine is needed to overcome the challenges of reaching vaccine potential recipients in rural and remote areas.

**Methods:**

We estimated the financial and economic costs of delivering HPV vaccination to ten-year-old girls at schools for the first vaccination cycle of the demonstration programme in the Manhiça district (southern Mozambique), delivered throughout 2014. We also estimated costs of an alternative scenario with a reduced number of doses and personnel, which was analogous to the second vaccination cycle delivered throughout 2015. Cost estimates followed a micro-costing approach and included interviews with key informants at different administrative levels through the administration of standard questionnaires developed by the World Health Organisation.

**Results:**

Considering only data from the first vaccination cycle (2014), which consisted in the administration of three doses, the average economic cost was US$17.59 per dose and US$52.29 per fully-immunised girl (FIG). Financial cost per dose (US$6.07) and per FIG (US$17.95) were substantially lower. The economic cost was US$15.53 per dose and US$31.14 per FIG when estimating an alternative cost scenario with reduced number of doses and personnel.

**Conclusions:**

The average economic cost per dose was lower than the ones recently reported for low- and middle-income countries. However, our estimation of the financial cost per FIG was higher than the ones observed elsewhere (ranging from US$2.49 in India to US$20.36 in Vietnam) due to the high percentage of out-of-school girls which, reduced vaccine coverage and, therefore, reduced the denominator. Due to budget constraints, if Mozambique is to implement nation-wide HPV vaccination targeted to ten-year-old girls at schools, a reduction in personnel costs should be operated either by restricting the outreach vaccinator team or the number of supervision visits.

**Electronic supplementary material:**

The online version of this article (10.1186/s12889-019-7338-4) contains supplementary material, which is available to authorized users.

## Background

Cervical cancer affected 570,000 women worldwide in 2018 and 311,000 died of the disease [1]. Cervical cancer is currently the most common type of cancer affecting women in low- and middle-income countries (LMICs) [2]. This is also the case in Mozambique, where 5,622 women were diagnosed with cervical cancer and 4,061 women died of the disease in 2017 [3]. Cervical cancer has also been identified to be the most common type of cancer between 1991 and 2008 in the country’s main Central Hospital [4]. However, due to underdiagnoses, any reported figure is very likely to be an underestimation of the real disease burden.

Human papillomavirus (HPV) is a necessary cause of cervical cancer [5]. Specifically, HPV 16 and 18 are the two genotypes responsible for about 70% of cervical cancer cases [6]. The development of safe and efficacious vaccines against the two high-risk HPV genotypes raised hopes of reducing the global incidence of cervical cancer. While most high-income countries progressively adopted publicly funded HPV vaccination programmes since 2006, few LMICs could afford its implementation due to the high vaccine price [7, 8]. To improve access in LMICs, an agreement to reduce HPV vaccines’ price to US$5 per dose took place in 2011 between manufacturers and the GAVI Vaccine Alliance, formerly known as the Global Alliance for Vaccination and Immunisation [9]. Only then, GAVI, national governments and other international organisations supported HPV vaccination programmes in LMICs with a yearly gross national income (GNI) per capita not higher than US$1,580 [10, 11].

In sub-Saharan Africa, preliminary estimates suggest that, under the assumption of high coverage and lifelong protection, the introduction of the vaccine is expected to result in high health gains in terms of both number of cancer cases and disability-adjusted life years (DALYs) averted [12]. However, even after high vaccine price reductions, one of the key issues associated with HPV vaccination is its cost of delivery and administration, particularly in rural and remote areas [13, 14]. Despite the efforts made, the unsubsidised cost of HPV vaccination is still higher than the majority of vaccines. Its delivery is challenging as the target group for HPV vaccination (girls between nine and 13 years of age) [6] differs markedly from the routine expanded programme on immunisation (EPI), which targets infants and young children [15].

The challenge of reaching the HPV vaccination target group is the absence of an entry point at health facility level, calling for the need to reach the girls outside the health system *stricto* *sensu*. Vaccination at schools has been suggested as an effective strategy to maximise both vaccine coverage [16] and the number of fully-immunised girls (FIGs) [17]. However, in LMICs some girls may be found absent from school on vaccination days, implying the need for establishing additional community strategies to maximise the reach [18]. These outreach approaches require high level of resources to identify the target population, engage them, train health professionals, teachers and other stakeholders, and finally deliver the vaccine [19–22].

In recognition of the high burden of cervical cancer in Mozambique, new efforts have been in place to reduce the disease burden in the country. Such efforts included a cervical cancer screening programme launched in 2009 [23], the consolidation of a cancer registry based at the Maputo Central Hospital [4], and the evaluation of introducing HPV vaccination to pre-adolescent girls as part of the national routine EPI by 2021. Under this framework, a demonstration programme was carried out in Mozambique consisting of vaccinating ten-year-old girls at schools, health facilities and communities in three districts: Manhiça, Manica and Mocímboa da Praia; south, centre and north of the country, respectively. The programme ran in two cycles, years 2014 and 2015, and was implemented as a three-dose schedule of Bivalent Cervarix® during the first year and a two-dose schedule during the second year; both providing a complete immunisation [24]. The programme implementation in Manhiça district followed a school-based approach and was funded and supported by GAVI as part of the initiative to introduce HPV vaccination in LMICs [25]. The Mozambican government funded the delivery in the two other districts [24]. This study estimated the costs associated with the demonstration programme of HPV vaccination during the 2014 cycle in the Manhiça district, and developed an alternative cost scenario for future implementation.

## Methods

### Study setting

The study was carried out in Manhiça, a semi-rural district 80 km north of Maputo city. With 229,539 inhabitants in 2013 [26], the population is unevenly distributed across the district with concentrations in the two main towns, Manhiça and Xinavane. There are some commercial activities on the main road crossing the district South-North [26], but most of the population lives in precarious dwellings and live of subsistence farming.

According to the National Institute of Statistics 92 primary schools and 2,974 girls ten years of age are present in the district [24, 26]. Manhiça encompasses one district and one rural hospital, 13 health facilities and one health post, covering the six administrative posts [26]. EPI services are available in 12 of the health facilities. Adjacent to the district hospital, the Manhiça Health Research Centre (in Portuguese Centro de Investigação em Saúde de Manhiça, CISM) operates a health and demographic surveillance system in the district, which monitors the entire population’s vital events at community level and records paediatric outpatients and hospital admissions since 1996 [27].

### Data collection approach

Primary data collection involved interviews with experts, referred to as key informants in this study, at the national, district (Manhiça) and sub-district levels (health facilities), to gather information on the use of resources during the first year demonstration programme [28]. Interviews with informants at different administrative levels allowed a better understanding of the overall vaccination process already in place and the relationships between central, district and local management of the vaccine procurement, delivery and administration. While representatives at the national level provided a wider view on both the current EPI state in Mozambique and on the challenges and resources needed for the potential inclusion of the new vaccine, local health professionals provided a day-to-day perspective of the use of resources. Data were complemented with specific information documented on the demonstration programme [24, 29], such as the number of schools and health facilities involved in the study, number of ten-year-old girls enrolled at school and registered in the district or data on drop-out rates, coverage and vaccination rounds.

Interviews were carried out face-to-face, unless not possible. If not possible, interviewees were contacted by phone or e-mail. Key representatives from the Ministry of Health (MoH) and Education (MoE) at central level were interviewed, including the current EPI Chief, EPI logistic head, EPI financial head, EPI data manager and the responsible of the Special Programmes Directorate in the MoE. At district level, the responsible of school health promotion at the Manhiça District Directorate of Education, a medical technician, a physician, the Chief Medical Officer at the Manhiça District Hospital, as well as representatives of CISM. Key implementation partners involved in the programme were included in the interviewees group; namely Village Reach, Fundação para o Desenvolvimento da Comunidade (FDC) and the United Nations International Children’s Emergency Fund (UNICEF).

Standard structured questionnaires developed by the World Health Organisation (WHO) were administered to interviewees [20]. Questionnaires used for data collection can be found in Additional file 1: S1. Questions inquired about the resources needed for the introduction and management of the new vaccine, including training and mobilisation campaigns, additional resources for the cold-chain and supervision, among others [30]. This standardised procedure allowed comparisons with other demonstration programmes held in other LMICs [21]. Data from questionnaires were incorporated in a spreadsheet prepared by the WHO for analytical and graphical data aggregation [30].

### Costing approach

The costs were initially retrieved in Mozambican meticais (MT) and later converted to US dollars (US$), using the official exchange rate published by the World Bank for the year 2014 (1US$ = 31.35MT) [31]. A linear depreciation approach was used to capture inflation and the value loss of certain products subject to devaluation, such as vehicles, using a standard discount rate of 3% per year [32].

The cost estimates for delivering HPV vaccination in Manhiça district through schools were based on a micro-costing approach in which all resources and costs associated to the purchase, storage, distribution and delivery the vaccine were quantified, including all indirect resources and costs related to those actions (such as, training and social mobilisation activities). Following an ingredient approach [32], every input was the result of the quantity of resource used multiplied by its correspondent unit cost [15].

Costs were differentiated between financial and economic [20]. While financial costs referred only to the use of resources paid by the MoH to execute the programme, economic costs also included the use of resources that did not necessarily imply a payment from the MoH. Thus, economic costs consisted of financial costs plus the use of resources already existing in the health system or donated by partners for the programme. Economic costs included: (1) the vaccine price, as it was subsidised by GAVI [19, 21]; (2) the use of cold-chain excess capacity; (3) the time of volunteers or other workers whose salary was not paid by the programme (considered as opportunity cost of time) [19, 21], and (4) capital costs already existing in the system and used during the programme, such as means of transport used for microplanning, training or service delivery. Moreover, distinction was done between incremental or full costs, when relevant [33]. While full cost analysis estimated the costs of all resources used for vaccine administration, independently from using already existing or not yet available resources, incremental analysis estimated the costs of all the additional resources not yet available to add a new vaccine, such as storage, transport or salaries.

Cold-chain needs were estimated according to available information on the current multi-tiered distribution system and the cold-chain management and needs [34, 35]. In 2014 the cold-chain infrastructure benefitted from excess capacity and no extra-investment was required for the sake of the demonstration project [35]. The use of existing excess capacities was estimated as the number of doses administered during the first vaccination cycle multiplied by the vaccine supply chain cost per dose estimated for a region in southern Mozambique [34]. The value of existing capacities was considered economic cost.

Costs were additionally categorised into introduction, recurrent, cold-chain supplement and other costs. Introduction costs, which occur at programme inception, included the value of resources required to start the implementation of the new vaccination. These costs consisted of (1) microplanning and training, (2) social mobilisation and information, education and communication (IEC) activities. Introduction costs were annualised over five years using a discount rate of 3%, as they were assumed to have a mid-term duration as part of vaccine implementation but only the first year amount was imputed to the programme costs of the 2014 cycle [15, 22]. Recurrent costs referred to the regular costs for vaccine delivery; such as (1) vaccine procurement, (2) service delivery, (3) supervision and monitoring and (4) other recurrent costs. The vaccine price, as part of the vaccine procurement cost, was considered economic and not financial cost [19], but custom clearance, syringes and safety boxes were considered both financial and economic costs.

Finally, estimated costs were expressed as average financial and economic cost per dose and per FIG. The average cost per dose was the sum of total costs divided by the sum of all doses delivered, which consisted of doses administered and the vaccine wastage. The average cost per FIG was computed as total introduction and cold-chain costs divided by the total target population plus total recurrent costs divided by the number of FIGs. The number of FIGs was defined as the sum of girls being administered three doses for the demonstration programme (2014 cycle) and two doses for the alternative scenario. Analytical expressions of the average cost per dose and FIG can be found in the Additional file 1: S2.

### Cost of alternative scenario

The programme was implemented over two vaccination cycles: lessons learnt in the first cycle (2014) helped improve the implementation of the second cycle (2015). Data on resources used were not collected over the second cycle. However, the second cycle inspired the estimate of resources needed for a potentially more efficient scenario, alternative to the first one, based on: (1) a reduction in the number of doses to fully-immunise a girl (from three to two), following WHO recommendations [36]; (2) a decrease in outreach and supervision teams to one vaccinator, one teacher and one supervisor; and (3) two community visits of outreach services for each health facility catchment area to increase vaccination coverage and reach girls absent from schools (by taking into account 12 health facilities in the district, a total 24 visits were assumed and the associated costs were considered as economic).

## Results

### The Manhiça demonstration programme

In total 20 key participants were contacted, of which 13 were interviewed and provided the inputs needed to assess the programme costs. The remaining seven were unavailable for interview. A list of interviewees can be found in the Additional file 1: Table S3. In Manhiça district the demonstration programme of HPV vaccination was executed by the MoH, using resources provided by GAVI following a school-based delivery strategy [24]. The MoH transferred some of the resources to the programme partners, such as CISM and FDC. Additional file 1: S4 details the role of the participant institutions.

UNICEF was the procurement agent for the HPV vaccine and equipment. Bivalent Cervarix® (two-dose vial) was the formulation used for the programme at a reduced procurement price of US$5.70 per dose. We added a 10% cost increase associated with custom clearance, such as boarding inspection, taxes or the transport cost to the central storage. While the vaccine price was fully covered by GAVI, costs related to custom clearance were paid by the MoH.

The main outputs of the programme implemented in Manhiça district are shown in Table 1. Three central level EPI supervisors were trained and assigned to oversee the programme implementation. This implied regular supervision visits from central to district level (US$64 *per diem*, per person). In total, 21 health professionals formed the active vaccination workforce in the district, with an average of 1.75 professionals per health facility catchment area to implement the programme during the first cycle (2014). However, two health professionals were assumed for calculation purposes to reflect the realistic scenario per health facility catchment area. Each health professional received US$48 *per diem* to deliver the vaccine at each school visit. According to district EPI data, the demonstration programme achieved 2,276 FIGs, 77% of the target population, and 6,945 doses were estimated to be administered throughout the 2014 cycle. The remaining 23% were girls who were not enrolled at school or were absent at vaccination time. The latter were informed by teachers that they could get vaccinated at health facilities. In the 2014 cycle, vaccination only occurred at schools. However, efforts were made to also reach and vaccinate girls at health facilities and at the community in case girls were not found at school. Despite such efforts, only two girls were tracked to get the vaccine at a health facility through an active search team in Maluana administrative post.

**Table 1:** Annual outputs of the human papillomavirus (HPV) demonstration programme in the Manhiça district for the 2014 cycle and the alternative scenario

Annual outputs	Demonstration Programme (2014 cycle)	Alternative scenario
# doses required to fully immunise a girl	3	2
# supervisors trained	3	1
# vaccinators per health facility	1	1
# vaccinators at schools	2	1
# start-up vaccination facility catchment area^a^	12	12
# start-up primary schools^b^	92	92
Number of fully-immunised girls (FIGs)^c^	2,276	2,791
Total doses of vaccine administered^c^	6,945	5,648
% of the total target population fully immunised	77%	94%

*Notes:*

^a^A total of 12 health facilities with EPI services were present in the district.

^b^92 schools according to the official list of MoE (Additional file 1: Table S5).

^c^Number of fully-immunised girls (FIGs) and doses obtained from district coverage data*.*

### Cost of the first HPV vaccination cycle implemented in Manhiça district

The total economic cost of the programme for the 2014 cycle was US$122,170 of which US$42,163 was financial. These amounts represent the total value of the resources used in the district of Manhiça in 2014, including resources used at national, provincial and district levels, and costs at health facilities and schools. Total amounts included vaccination at schools with an achieved coverage of 77%. No improvement in cold-chain capacities was required to implement the programme in Manhiça due to existing space capacity.

**Fig. 1:**
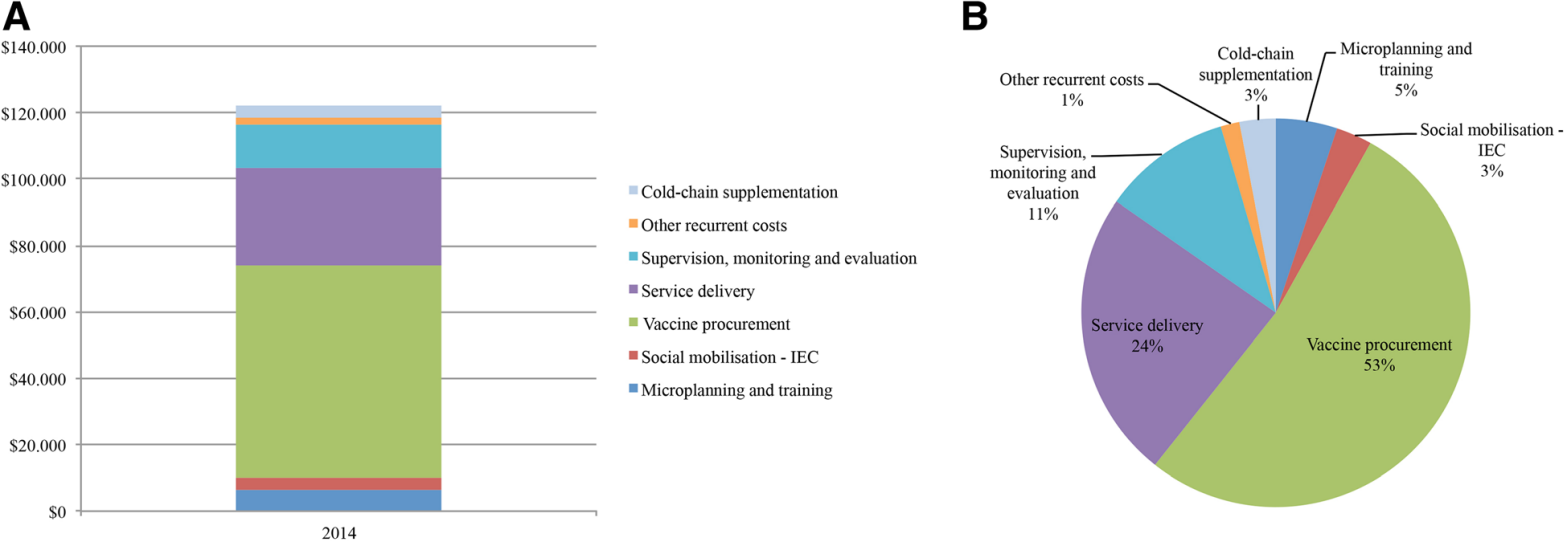
Economic costs of the human papillomavirus (HPV) vaccination programme (2014 cycle) in the Manhiça district expressed as (**a**) US$ and (**b**) percentages

Table 2 shows the total financial and economic costs of the HPV vaccination programme for the 2014 cycle. Concerning financial costs, most of the resources used were recurrent costs (US$36,577) and consisted of vaccine procurement (US$12,774), service delivery (US$8,804), monitoring and evaluation (US$13,086) and waste management (US$1,914). Introduction financial costs attributable to the 2014 cycle were US$5,585 and consisted of microplanning and training activities (US$2,484) and social mobilisation and IEC activities (US$3,101). When considering economic costs, the total cost of the programme reached US$122,170. This increase (US$80,007) was mainly explained by adding the vaccine price (US$51,463) to the procurement economic cost (US$64,237) and the use of existing cold-chain excess capacity in the district (US$3,681). The increase in the economic costs of service delivery (US$20,563), microplanning and training activities (US$3,748) and social mobilisation and IEC activities (US$552) was due to the opportunity cost of labour and vehicles already existing in the health system. A graphical representation of the economic costs is shown in Fig. 1. Vaccine procurement represented up to 53% of the total economic cost, being the largest recurrent and total economic cost of the programme. Service delivery reached 24% of the total economic cost, while monitoring and evaluation represented 11%. Microplanning and training contributed 5%; social mobilisation and IEC activities and cold-chain utilisation of excess capacity contributed 3% each to the total. Other recurrent economic costs (1%) completed the programme disbursements, consisting of the waste management.

**Table 2:** Financial and economic costs of the 2014 vaccination cycle (US$)

	FINANCIAL COSTS		ECONOMIC COSTS	
Introduction costs^a^				
Microplanning and training	2,484	5.89%	6,232	5.10%
Social mobilisation – IEC^b^	3,101	7.36%	3,653	2.99%
Subtotal introduction costs	5,585	13.25%	9,885	8.09%
Recurrent costs				
Vaccine procurement^c^	12,774	30.30%	64,237	52.58%
Service delivery	8,804	20.88%	29,367	24.04%
Supervision, monitoring & evaluation	13,086	31.04%	13,086	10.71%
Other recurrent costs	1,914	4.54%	1,914	1.57%
Subtotal recurrent costs	36,577	86.75%	108,604	88.90%
Cold-chain supplement^c^				
Subtotal cold-chain supplement	0	0.00%	3,681	3.01%
Total costs	42,163		122,170	

*Note:*

^a^Introduction costs were annualised in five years and only the first year amount was attributable for the 2014 vaccination cycle

^b^*IEC* information, education and communication

^c^The total vaccine procurement economic cost (US$64,237) was the sum of custom clearance, syringes and safety boxes costs (US$12,774), considered both financial and economic costs, and the vaccine price (US$56,610), treated as economic cost. The use of existing cold-chain excess capacity (US$3,681) was considered economic cost. Other economic costs were capital and labour costs, which incurred in opportunity costs

Table 3 shows the financial and economic cost per dose and per FIG. The average economic cost delivered was US$17.59 per dose (US$6.07 financial cost per dose), of which US$1.42 corresponded to introduction, US$15.64 to recurrent and US$0.53 to cold-chain economic cost. The demonstration programme (cycle 2014) implied an average economic cost of US$52.29 per FIG (US$17.95 financial cost per FIG).

**Table 3:** Average financial and economic costs per dose and fully-immunised girl (FIG) for the demonstration programme (2014 cycle) and the alternative scenario (US$)

	Financial Cost				Economic Cost			
	Introduction cost	Recurrent cost	Cold-chain	Total cost	Introduction cost	Recurrent cost	Cold-chain	Total cost
Demonstration Programme (2014 cycle)								
Cost per dose	0.80	5.27	0.00	6.07	1.42	15.64	0.53	17.59
Cost per FIG	1.88	16.07	0.00	17.95	3.32	47.73	1.24	52.29
Alternative scenario								
Cost per dose	0.99	4.01	0.00	5.00	1.75	13.25	0.53	15.53
Cost per FIG	1.88	8.11	0.00	9.99	3.32	26.81	1.01	31.14

### Costs of the alternative scenario

We estimated the programme cost if the number of doses were reduced to two; the supervision team to one supervisor and the outreach team at schools to one vaccinator and one teacher; and if two community outreach visits were carried out per health facility catchment area, in order to increase the coverage of FIGs to 94% of the target population (Table 1). This set of changes implied a reduction in the vaccine procurement economic cost from US$64,237 to US$52,241. It also implied a reduction in the service delivery economic cost from US$29,367 to US$13,283, as well as supervision cost from US$13,086 to US$7,401 as the reduction of supervisors compensated the community outreach costs. All in all, the programme economic cost would reduce to US$87,718 (US$28,223 financial costs). Financial and economic costs of the alternative scenario can be found in the Additional file 1: Table S6.

The average economic cost reduced to US$15.53 per dose (US$5.00 financial cost per dose), US$1.75 corresponding to introduction, US$13.25 to recurrent and US$0.53 to cold-chain economic costs. The average economic and financial cost per FIG reduced to US$31.14 and US$9.99 respectively (Table 3).

## Discussion

During the first vaccination cycle of the demonstration programme in Manhiça district, when three doses were administered, the average financial cost per dose was US$6.07 (US$17.59 economic). Our estimations were below the mean financial (US$8.74) and economic cost per dose (US$19.98) recently calculated in a study reporting the findings of 12 demonstration projects in different LMICs, ten of which were sub-Saharan African countries [19]. However, our estimations of the average financial (US$17.95) and economic (US$52.29) cost per FIG were higher than most of those obtained by other published studies reporting estimates of similar HPV vaccine demonstration programmes in LMICs [15, 20–22].

Nonetheless, cross-country comparisons should be made with caution. First, our estimations refer to the Manhiça demonstration programme and no scale-up projection for the introduction of the vaccine at a national level was computed due to limited data on key variables, such as reliable target population and number of schools. Second, there is a great cost variability across countries [21]: the average financial cost per FIG ranged from US$2.49 in India, under a school-based delivery strategy and community outreach services, to US$20.36 in Vietnam, also under a school-based delivery strategy. However, two pilot projects evaluated in Tanzania are likely to be more comparable to the programme in Manhiça: population size and density were similar, both followed a school-based delivery strategy, achieved similar coverage levels to that in Manhiça (79 and 72%) and resulted in similar average financial cost per FIG (US$15.27 and US$19.17) but higher average economic costs per FIG (US$66 to US$78) due to higher transport and storage costs [21, 22].

The high average financial cost per FIG in Manhiça (US$17.95) is mainly explained by the lower coverage rate achieved (77%) than most of other demonstration programmes, and by certain country characteristics, such as: (1) low population density [19], (2) long distance between health facilities and schools, and (3) programme design with high intensity of resources devoted to the programme [19].

The school-based delivery strategy has important implications in terms of coverage that critically affects the cost per FIG in low-income countries: (1) school-based vaccine programmes face poor planning, and (2) high percentage of school absenteeism and out-of-school girls are commonly reported [13]. According to the Mozambican National Institute of Statistics, 2,974 ten-year-old girls lived in Manhiça district at the time of the first cycle [24], but only 2,280 were enrolled at schools according to the school census. It implies that 694 girls (23.34% of the target population) could not be reached following a school-based approach, therefore reducing the coverage and increasing the cost per FIG. One possibility is to increase the coverage through community visits of outreach services to reach girls in remote settings [37]. Assuming two community visits per health facility catchment area, and under a three-dose regime, the average economic cost per dose would decrease to US$17.11 as coverage increases (94%). However, the average economic cost per FIG would still remain high (US$52.03) due to the increased number of vaccines administered: procurement of US$77.52 and delivery of US$36.35. Alternatively, a reduction in the supervision team to one and the outreach team at schools to one vaccinator and one teacher would diminish the total cost to US$101,629 (17%) due to halving the vaccine delivery cost to US$14,683 and reducing supervision to US$7,218, allowing a reduction in the average financial cost per FIG to US$13.44 (US$43.26 economic cost). Finally, should the two-doses regimen have been implemented during the 2014 cycle, it would have allowed reducing the overall economic cost of the programme (US$90,128; 26%) due to reductions in the vaccine procurement (US$43,190; 32.76%) and delivery (US$19,578; 33.33%), yielding to a decrease in the average economic cost per FIG to US$37.89, thanks to savings in number of school visits. A combination of these three measures would reduce the average economic cost to US$15.53 per dose (US$5.00 financial) and to US$31.14 per FIG (US$9.99 financial).

Demonstration programmes in LMICs have been reported to be resource-intensive, but national vaccine implementation should involve substantial economies of scales [19]. The Mozambican MoH projected an HPV vaccination cost of US$39 per FIG through the routine EPI schedule, lower than the one obtained in our study [38]. However, it is important to note that Manhiça is dissimilar to other districts, due to its atypical socio-economic characteristics and circumstances related to its healthcare network privileged by the presence of a research institution. Thus, if Mozambique is to implement HPV vaccination targeted at ten-year-old girls in schools with minimal cold-chain requirements fulfilled, cuts must be made to the use of resources. First, reduction in the outreach team to one vaccinator and one teacher should be considered. Second, the need for subsistence support to the team as well as constant supervisory visits should be revisited. Further, despite the EPI, the MoH will most likely rely on implementation partners to support training, IEC and transport of the brigades in the field, and approaches to sustainability should be addressed.

The evaluation of alternative delivery mechanisms of HPV vaccination either under a health facility delivery strategy or as part of existing programmes (adolescent health programmes) would reveal which strategies are most efficient and less costly in the Mozambican context [39, 40].

## Conclusion

The estimation of the demonstration programme to deliver HPV vaccination in Manhiça reached an average financial cost of US$6.07 per dose and of US$17.95 per FIG (economic cost of US$17.59 per dose and of US$52.29 per FIG). The cost per dose was below the one reported from similar demonstration programmes in other LMICs but the cost per FIG was among the highest as out-of-school girls limited vaccination coverage. However, the financial cost per FIG could be reduced to US$9.99 (US$31.14 economic) under a two-dose schedule, combined with an improvement in vaccine coverage and reductions in personnel costs. Nonetheless, the vaccine preventive effect might sharply reduce the number of cervical cancer cases in Mozambique despite the high costs and challenges of a national scale-up of the intervention.

## Additional file

Additional file 1: Study collection forms as S1, analytical expressions of the average cost per dose and FIG as S2, list of interviewees as Table S3, role of participant institutions as S4, number of schools and girls in the district as Table S5 and financial and economic costs of the alternative scenario as Table S6. (PDF 2056 kb)

## Data Availability

The datasets used and/or analysed during the current study are available from the corresponding author on reasonable request.

## Abbreviations

CISM: Centro de Investigação em Saúde de Manhiça
DALY: Disability-adjusted life year
EPI: Expanded programme on immunisation
FDC: Fundação para o Desenvolvimento da Comunidade
FIG: Fully immunised girl
GAVI: Global Alliance for Vaccines and Immunisations
GNI: Gross national income
HPV: Human papillomavirus
IEC: Information, education and communication
LMIC: Low- and middle-income country
MoE: Ministry of Education
MoH: Ministry of Health
UNICEF: United Nations International Children’s Emergency Fund
WHO: World Health Organisation
