# Supplementary material for: Costs associated with delivering HPV vaccination in the context of the first year demonstration programme in southern Mozambique
Source: BMC Public Health. 2019 Aug 1;19:1031. doi: 10.1186/s12889-019-7338-4 (PMC6676560; doi:10.1186/s12889-019-7338-4)
Supplement: Supplementary file 1 — Study collection forms as S1, analytical expressions of the average cost per dose and FIG as S2, list of interviewees as Table S3, role of participant institutions as S4, number of schools and girls in the district as Table S5 and financial and economic costs of the alternative scenario as Table S6. (PDF 2056 kb) [file 12889_2019_7338_MOESM1_ESM.pdf]

Supplementary file

Appendix S1. C4P Demo Collection form.

See next page.

C4P Demo Collection form

Form 1: National Level

A/ General Country information

Country information		
Country name		LABELS
Country currency		DASHBOARD
Exchange rate (Local currency to $US)		ECON&DEMOG INPUTS
Inflation rate (%)		ECON&DEMOG INPUTS
Annual discount rate (%)		ECON&DEMOG INPUTS
Estimated useful years for introduction costs		ECON&DEMOG INPUTS
Annual population growth rate (%)	cMYP	ECON&DEMOG INPUTS
Proportion of target population in school		ECON&DEMOG INPUTS
Proportion of target population out of school		ECON&DEMOG INPUTS
Project demonstration start year		LABELS

Country administrative levels		
First level label		LABELS
Second level label		LABELS
Third level label		LABELS
Fourth level label		LABELS
Facility Level name		LABELS
School type level name		LABELS

B/ Demo Project location

District A		District B		
District A name		District B name		SUBNATIONAL INFO
# of sub districts		# of sub districts		SUBNATIONAL INFO
Estimated target population		Estimated target population		SUBNATIONAL INFO
# of vaccination facilities		# of vaccination facilities		SUBNATIONAL INFO
# of health workers in vaccination facilities		# of health workers in vaccination facilities		SUBNATIONAL INFO
# of primary school		# of primary school		SUBNATIONAL INFO

Notes

C4P Demo Collection form

C/ Vaccine delivery Strategy

⊕ How is the vaccine being delivered? (you can choose a single or a combination of methods).

	Through schools (vaccinators go out)	If ☒ then, # to visit to a school per year	Through Health Facilities (Patients come in)	Through Outreach	If ☒ then, # of outreach per year (other than to a school)	
**District A**	☐		☐	☐		STRATEGY
**District B**	☐		☐	☐		STRATEGY

⊕ Provide expected coverage and dropout rates per delivery methods.

**District A**	Through schools (vaccinators go out)	Through Health Facilities (Patients come in)	Through Outreach	
D1 coverage rate	%	%	%	STRATEGY
Dropout rate (between D1 and D2)	%	%	%	STRATEGY
Dropout rate (between D2 and D3)	%	%	%	STRATEGY

**District B**	Through schools (vaccinators go out)	Through Health Facilities (Patients come in)	Through Outreach	
D1 coverage rate	%	%	%	STRATEGY
Dropout rate (between D1 and D2)	%	%	%	STRATEGY
Dropout rate (between D2 and D3)	%	%	%	STRATEGY

	**District A**	**District B**	
Average # of vaccinators per school visit			SERVICE DELIVERY
Average # of teachers assisting per school visit			SERVICE DELIVERY
Average # of vaccinators per outreach activity			SERVICE DELIVERY
Average # of minutes per person vaccinated in Health facility			SERVICE DELIVERY
Average # of minutes per person vaccinated during a school visit			SERVICE DELIVERY
Average # of minutes per person vaccinated in other outreach activity			SERVICE DELIVERY
Average length (in days) per school visit			SERVICE DELIVERY
Average length (in days) per other outreach visit			SERVICE DELIVERY

	**District A**		**District B**		
	Unit Cost (Financial)	Unit Cost (Economic)	Unit Cost (Financial)	Unit Cost (Economic)	SERVICE DELIVERY
Average monthly salary and benefits of a health facility vaccinator					SERVICE DELIVERY
Average cost of a R/T transport for a school vaccination visit (per person)					SERVICE DELIVERY
Average cost of a R/T transport for an other outreach visit (per person)					SERVICE DELIVERY
Average per diem for outreach (out of station) visits by a vaccinator					SERVICE DELIVERY
Average per diem allowance for a teacher assisting with school vaccination					SERVICE DELIVERY

C4P Demo Collection form

D/ Vaccine information

Type and presentation of HPV vaccine used				
☐ Quadravalent Gardasil (1 dose vial)*1	☐ Bivalent Cervarix (1 dose vial)*1	☐ Bivalent Cervarix (1 dose vial)*100	☐ Bivalent Cervarix (2 dose vial)*10	COLD CHAIN
☐ Quadravalent Gardasil (1 dose vial)*10	☐ Bivalent Cervarix (1 dose vial)*10	☐ Bivalent Cervarix (2 dose vial)*1	☐ Bivalent Cervarix (2 dose vial)*100	

Planned doses to give to each FIG	
	VACCINES

Vaccine cost	Vaccine Dose	AD Injection Syringe	Safety box	
Unit cost ($)	cMYP	cMYP	cMYP	VACCINES
Subsidy ($)	cMYP	cMYP	cMYP	VACCINES

Additional charges to vaccine cost (%)		
Marine Insurance	%	PLUG-VACS
Wharfage	%	PLUG-VACS
Handling	%	PLUG-VACS
Removal from transport	%	PLUG-VACS
Port storage	%	PLUG-VACS
Transport to stock management centre	%	PLUG-VACS
Bank charges	%	PLUG-VACS
Destination inspection fee	%	PLUG-VACS
Storage in warehouses (cold rooms, refrigeration and insurance coverage)	%	PLUG-VACS
Distribution to zonal centres and insurance coverage	%	PLUG-VACS

	Vaccine	Syringe	Safety Box	
Assumed wastage rate (%)	cMYP	cMYP	cMYP	VACCINES
Planned buffer/reserve stock (%)	cMYP	cMYP	cMYP	VACCINES

Capacity of safety box (# syringes)	
	VACCINES

Notes

E/ Micro planning activities

For detailed calculation, refer to form 4.

	Number	Financial Cost	Economic Cost	
Micro planning activities at First level				MICROPLANNING
Micro planning activities at second level				MICROPLANNING
Micro planning activities at third level				MICROPLANNING

C4P Demo Collection form

F/ Training activities – *For detailed calculation, refer to form 4.*

Max # of participants per training of trainers workshop		TRAINING
Max # of participants per training of supervisors		TRAINING
Max # of participants per training of vaccinators		TRAINING
# of trainers trained per district		TRAINING
# of supervisors trained per Sub district		TRAINING
# of vaccinators trained per vaccination facility		TRAINING

Notes

	Unit Cost (Financial)	Unit Cost (Economic)	
Curriculum Development workshop			TRAINING
Training of Trainers workshop			TRAINING
Training of supervisors workshop			TRAINING
Training of vaccinators workshop			TRAINING

G/ IEC and Social Mobilization– *For detailed calculation, refer to form 4.*

	#	Unit Cost (Financial)	Unit Cost (Economic)	
Sensitization event at first level				SOCIALMOB-IEC
Sensitization event per project area				SOCIALMOB-IEC
Sensitization event per sub project area				SOCIALMOB-IEC
Sensitization event per facility catchment area				SOCIALMOB-IEC
Sensitization event per school				SOCIALMOB-IEC

	Unit Cost (Financial)	Unit Cost (Economic)	
Initial IEC support Country level			SOCIALMOB-IEC
Initial IEC support Project area level			SOCIALMOB-IEC
Initial IEC support Sub project area level			SOCIALMOB-IEC
Initial IEC support Facility catchment area level			SOCIALMOB-IEC
Initial IEC support School level			SOCIALMOB-IEC

**H/ Supervision and monitoring**	#	Unit Cost (Financial)	Unit Cost (Economic)	
Supervision visit from first level				MONITORING
Supervision visit from second level				MONITORING
Supervision visit from third level				MONITORING
Monitoring record book per facility per year (accounting wastage)				MONITORING
Vaccination tally sheet reporting forms per facility per year (accounting wastage)				MONITORING
Vaccination cards per HPV-1 (accounting for wastage)				MONITORING
Post Introduction Evaluation				MONITORING

Proportion of supervisory cost allocated to HPV vaccine	%	MONITORING

C4P Demo Collection form

I/ Cold chain (supplement)

Number of supply/resupply shipments per year		
From Manufacturer to Central stores		COLD CHAIN
From Central stores to intermediate stores		COLD CHAIN
From intermediate stores to health facility		COLD CHAIN

	Estimate excess cold chain capacity available at each level	Estimate expenditure required to purchase additional space needed	
Central stores level	cm^3	$US	COLD CHAIN
Intermediate stores level	cm^3	$US	COLD CHAIN
Health facility level	cm^3	$US	COLD CHAIN

Estimate expenditure required to purchase additional space needed		
Central stores level	$	COLD CHAIN
Intermediate stores level	$	COLD CHAIN
Health facility level	$	COLD CHAIN

Useful life years for cold chain storage supplement		
Central stores equipment (assume cold rooms)	___ ULY	COLD CHAIN
Intermediate stores equipment (assume cold rooms)	___ ULY	COLD CHAIN
Health facility equipment (assume refrigerator)	___ ULY	COLD CHAIN

J/ Other costs

⊕ List in the table below all additional recurrent costs per dose administered:

Other recurrent cost item	Unit Cost (Financial)	Unit Cost (Economic)	
…			OTHER
…			OTHER
…			OTHER

⊕ List in the table below all additional Capital goods required for the demo project:

Other Capital goods	# needed	Unit Cost (Financial)	Useful life years	
…				OTHER
…				OTHER
…				OTHER

Notes

C4P Demo Collection form
Form 2: District Level

A/ Demo project location

**District name**		SUBNATIONAL INFO
Number of sub districts		SUBNATIONAL INFO
Estimated target population		SUBNATIONAL INFO
# of vaccination facilities		SUBNATIONAL INFO
# of health workers in vaccination facilities		SUBNATIONAL INFO
# of primary school		SUBNATIONAL INFO
Proportion of target population in school		ECON&DEMOG INPUTS
Proportion of target population out of school		ECON&DEMOG INPUTS
Project demonstration start year		LABELS

B/ Vaccine delivery Strategy

How is the vaccine being delivered? (you can choose a single or a combination of methods).

Through schools (vaccinators go out)	If ☒ then, # to visit to a school per year	Through Health Facilities (Patients come in)	Through Outreach	If ☒ then, # of outreach per year (other than to a school)	
☐		☐	☐		STRATEGY

Provide expected coverage and dropout rates per delivery methods.

	Through schools (vaccinators go out)	Through Health Facilities (Patients come in)	Through Outreach	
D1 coverage rate	%	%	%	STRATEGY
Dropout rate (between D1 and D2)	%	%	%	STRATEGY
Dropout rate (between D2 and D3)	%	%	%	STRATEGY

Average # of vaccinators per school visit		SERVICE DELIVERY
Average # of teachers assisting per school visit		SERVICE DELIVERY
Average # of vaccinators per outreach activity		SERVICE DELIVERY
Average # of minutes per person vaccinated in Health facility		SERVICE DELIVERY
Average # of minutes per person vaccinated during a school visit		SERVICE DELIVERY
Average # of minutes per person vaccinated in other outreach activity		SERVICE DELIVERY
Average length (in days) per school visit		SERVICE DELIVERY
Average length (in days) per other outreach visit		SERVICE DELIVERY

	Unit Cost (Financial)	Unit Cost (Economic)	
Average monthly salary and benefits of a health facility vaccinator			SERVICE DELIVERY
Average cost of a R/T transport for a school vaccination visit (per person)			SERVICE DELIVERY
Average cost of a R/T transport for an other outreach visit (per person)			SERVICE DELIVERY
Average per diem for outreach (out of station) visits by a vaccinator			SERVICE DELIVERY
Average per diem allowance for a teacher assisting with school vaccination			SERVICE DELIVERY

C4P Demo Collection form

C/ Micro planning activities - *For detailed calculation, refer to form 4.*

	Number	Financial Cost	Economic Cost	
Micro planning activities at second level				MICROPLANNING
Micro planning activities at third level				MICROPLANNING

D/ Training activities - *For detailed calculation, refer to form 4.*

# of trainers trained per district		TRAINING
# of supervisors trained per Sub district		TRAINING
# of vaccinators trained per vaccination facility		TRAINING

	Unit Cost (Financial)	Unit Cost (Economic)	
Curriculum Development workshop			TRAINING
Training of Trainers workshop			TRAINING
Training of supervisors workshop			TRAINING
Training of vaccinators workshop			TRAINING

E/ IEC and Social Mobilization– *For detailed calculation, refer to form 4.*

	#	Unit Cost (Financial)	Unit Cost (Economic)	
Sensitization event per project area				SOCIALMOB-IEC
Sensitization event per sub project area				SOCIALMOB-IEC
Sensitization event per facility catchment area				SOCIALMOB-IEC
Sensitization event per school				SOCIALMOB-IEC

	Unit Cost (Financial)	Unit Cost (Economic)	
Initial IEC support Project area level			SOCIALMOB-IEC
Initial IEC support Sub project area level			SOCIALMOB-IEC
Initial IEC support Facility catchment area level			SOCIALMOB-IEC
Initial IEC support School level			SOCIALMOB-IEC

F/ Supervision and monitoring

	Number	Unit Cost (Financial)	Unit Cost (Economic)	
Supervision visit from second level				MONITORIN
Supervision visit from third level				MONITORIN
Monitoring record book per facility per year (accounting wastage)				MONITORIN
Vaccination tally sheet reporting forms per facility per year (accounting wastage)				MONITORIN
Vaccination cards per HPV-1 (accounting for wastage)				MONITORIN
Post Introduction Evaluation				MONITORIN

Proportion of supervisory cost allocated to HPV vaccine	%	MONITORING

C4P Demo Collection form

G/ Cold chain (supplement)

Number of supply/resupply shipments per year		
From Central stores to intermediate stores		COLD CHAIN
From intermediate stores to health facility		COLD CHAIN

	Estimate excess cold chain capacity available at each level	Estimate expenditure required to purchase additional space needed	
Intermediate stores level	cm^3	$US	COLD CHAIN
Health facility level	cm^3	$US	COLD CHAIN

Estimate expenditure required to purchase additional space needed		
Intermediate stores level	$	COLD CHAIN
Health facility level	$	COLD CHAIN

Useful life years for cold chain storage supplement		
Intermediate stores equipment (assume cold rooms)	ULY	COLD CHAIN
Health facility equipment (assume refrigerator)	ULY	COLD CHAIN

H/ Other costs

List in the table below all additional recurrent costs per dose administered:

Other recurrent cost item	Unit Cost (Financial)	Unit Cost (Economic)	
…			OTHER
…			OTHER
…			OTHER

List in the table below all additional Capital goods required for the demo project:

Other Capital goods	# needed	Unit Cost (Financial)	Useful life years	
…				OTHER
…				OTHER
…				OTHER

Notes

C4P Demo Collection form
Form 3: Vaccine Delivery Level

A/ Health catchment area information

**Health facility name**		
Proportion of target population in school		ECON&DEMOG INPUTS
Proportion of target population out of school		ECON&DEMOG INPUTS
# of health workers in vaccination facilities		SUBNATIONAL INFO
# of primary school		SUBNATIONAL INFO

B/ Vaccine delivery Strategy

How is the vaccine being delivered? (you can choose a single or a combination of methods).

	Through schools (vaccinators go out)	If ☒ then, # to visit to a school per year	Through Health Facilities (Patients come in)	Through Outreach	If ☒ then, # of outreach per year (other than to a school)	
**Health catchment area**	☐		☐	☐		STRATEGY

Provide expected coverage and dropout rates per delivery methods:

**Health catchment area**	Through schools (vaccinators go out)	Through Health Facilities (Patients come in)	Through Outreach	
D1 coverage rate	%	%	%	STRATEGY
Dropout rate (between D1 and D2)	%	%	%	STRATEGY
Dropout rate (between D2 and D3)	%	%	%	STRATEGY

	Health catchment area	
Average # of vaccinators per school visit		SERVICE DELIVERY
Average # of teachers assisting per school visit		SERVICE DELIVERY
Average # of vaccinators per outreach activity		SERVICE DELIVERY
Average # of minutes per person vaccinated in Health facility		SERVICE DELIVERY
Average # of minutes per person vaccinated during a school visit		SERVICE DELIVERY
Average # of minutes per person vaccinated in other outreach activity		SERVICE DELIVERY
Average length (in days) per school visit		SERVICE DELIVERY
Average length (in days) per other outreach visit		SERVICE DELIVERY

	Unit Cost (Financial)	Unit Cost (Economic)	
Average monthly salary and benefits of a health facility vaccinator			SERVICE DELIVERY
Average cost of a R/T transport for a school vaccination visit (per person)			SERVICE DELIVERY
Average cost of a R/T transport for an other outreach visit (per person)			SERVICE DELIVERY
Average per diem for outreach (out of station) visits by a vaccinator			SERVICE DELIVERY
Average per diem allowance for a teacher assisting with school vaccination			SERVICE DELIVERY

C4P Demo Collection form

C/ Training and supervision

# of vaccinators trained per vaccination facility		TRAINING

Supervision visit from third level				MONITORING
Monitoring record book per facility per year (accounting wastage)				MONITORING
Vaccination tally sheet reporting forms per facility per year (accounting wastage)				MONITORING

Proportion of supervisory cost allocated to HPV vaccine	%	MONITORING

	Estimate excess cold chain capacity available at each level	Estimate expenditure required to purchase additional space needed	
Health facility level	cm^3	$US	COLD CHAIN

Estimate expenditure required to purchase additional space needed		
Health facility level	$	COLD CHAIN

G/ IEC and Social Mobilization– *For detailed calculation, refer to form 4.*

	#	Unit Cost (Financial)	Unit Cost (Economic)	
Sensitization event per facility catchment area				SOCIALMOB-IEC
Sensitization event per school				SOCIALMOB-IEC

	Unit Cost (Financial)	Unit Cost (Economic)	
Initial IEC support Facility catchment area level			SOCIALMOB-IEC
Initial IEC support School level			SOCIALMOB-IEC

H/ Supervision and monitoring	#	Unit Cost (Financial)	Unit Cost (Economic)	
Supervision visit from first level				MONITORING
Supervision visit from second level				MONITORING
Supervision visit from third level				MONITORING
Monitoring record book per facility per year (accounting wastage)				MONITORING
Vaccination tally sheet reporting forms per facility per year (accounting wastage)				MONITORING
Vaccination cards per HPV-1 (accounting for wastage)				MONITORING
Post Introduction Evaluation				MONITORING

Proportion of supervisory cost allocated to HPV vaccine	%	MONITORING

Notes

C4P Demo Collection form

I/ Cold chain (supplement)

Number of supply/resupply shipments per year		
From intermediate stores to health facility		COLD CHAIN

	Estimate excess cold chain capacity available at each level	Estimate expenditure required to purchase additional space needed	
Health facility level	cm^3	$US	COLD CHAIN

Estimate expenditure required to purchase additional space needed		
Health facility level	$	COLD CHAIN

Useful life years for cold chain storage supplement		
Health facility equipment (assume refrigerator)	__ ULY	COLD CHAIN

J/ Other costs

List in the table below all additional recurrent costs per dose administered:

Other recurrent cost item	Unit Cost (Financial)	Unit Cost (Economic)	
...			OTHER
...			OTHER
...			OTHER

List in the table below all additional Capital goods required for the demo project:

Other Capital goods	# needed	Unit Cost (Financial)	Useful life years	
...				OTHER
...				OTHER
...				OTHER

Notes

C4P Demo Collection form

PLUG-MICRO
PLUG-TRAIN
PLUG-MEETING

Form 4: Micro planning and training activities - *Detailed calculation*

Use this form to detail the cost of micro planning, training and social mobilization (sensititzation) events. Use a different form for each different event.

Personnel (government and salaried staff only)

Category	Cost item	# of Persons	# of Days	Unit cost
Trainers & facilitators	Trainers			
	Facilitators			
Resource personnel				
Support personnel				
Participants				

Transport and travel cost items

Category	Cost item	# of Persons	# of Days	Unit cost
Per diems				
Transport allowances				
Other allowances				

Other direct cost items

External staff (contract hire)				
Category	Cost item	# of Rooms	# of Nights	Unit cost
Lodging				
Category	Cost item	# of Rooms	# of Days	Unit cost
Hall rental				
Category	Cost item	# of Persons	# of Days	Unit cost
Meals	Lunch			
	Dinner			
Refreshments	Morning break			
	Afternoon break			
Supplies	Daily supplies			
Category	Cost item	# of Persons	# of Materials	Unit cost
Course meeting materials				
Category	Cost item	# of Pieces	# of Days	Unit cost
Equipment (rental)				
Other (specifiy)				

Appendix S2. Analytical expressions to compute the average: (1) cost per dose and (2) cost per fully-immunised girl (FIG)

$$\textbf{\textit{average cost per dose}} = \sum(\textit{introduction costs for the } 1st \textit{ year} + \textit{recurrent costs} + \textit{cold chain costs}) / \textit{number of doses delivered};$$

being:

$$\textit{introduction costs} = \textit{microplanning costs} + \textit{training costs} + \textit{IEC} \,\&\, \textit{social mobilization costs};$$

$$\textit{recurrent costs} = \textit{vaccine procurement cost} + \textit{vaccine delivery cost} + \textit{supervision} \,\&\, \textit{monitoring costs} + \textit{other costs};$$

$$\textbf{\textit{average cost per FIG}} = \sum(\textit{introduction costs for the } 1st \textit{ year} + \textit{cold chain costs}) / \textit{target population} + \sum \textit{recurrent costs} / \textit{number of FIG};$$

being:

$$\textit{target population} = \textit{number of } 10 \textit{ years old girls in Manhiça district}.$$

Table S3. List of interviewees according to their role in process

Institution	Position	Contacted and interviewed	Contacted but not interviewed
CISM	Financial manager	YES	
Manhiça District Hospital	Medical technician	YES	
Manhiça District Hospital	Physician	YES	
Manhiça District Hospital	Physician chief	YES	
Direcção Distrital Educação Manhiça	Health promotion responsible	YES	
Ministry of Education	Programas Especiais responsible	YES	
CMAM	Deputy director		YES
Ministry of Health	Current EPI chief	YES	
Ministry of Health	Former EPI chief		YES
Ministry of Health	Financial EPI head	YES	
Ministry of Health	Logistics EPI head	YES	
Ministry of Health	Data manager	YES	
Ministry of Health	Supervisor in HPV demo		YES
Ministry of Health	Supervisor in HPV demo		YES
Direcção Provincial Saúde Maputo	Provincial EPI responsible		YES
WHO	Involved in HPV project		YES
UNICEF	Involved in HPV project	YES	
Village Reach	Involved in HPV project	YES	
USAID	Involved in HPV project		YES
FDC	Involved in HPV project	YES	

Annex S4. Role of the programme partners

Role of the Ministry of Education

The District Directorate of Education (DDE) did not receive direct financial support from the Ministry of Health (MoH) to implement the human papillomavirus (HPV) programme in the district. In preparation for the campaign, its task consisted of coordinating the identification of 10-year-old girls with the support of the Centro de Investigação em Saúde de Manhiça (CISM). During this process, teachers used the girls name as personal identification and recorded the information in registration books at each school, censing the target population. On the one hand, the school-based census allowed achieving the vaccination of almost all girls enrolled at school (table S2.1). On the other hand, this approach did not allow reaching out-of-school girls in the community. Manhiça DDE made an effort to actively participate in the capacity building, which took place prior to the start of each vaccination cycle and targeted at both health and education professionals. Discussions at central level resulted in the recommendation of cascade training targeting the Pedagogic Influence Zones, which were in total 17. One or two DDE representatives were supervising the course of the programme during 7 days (65 US$ *per diem*), implying the use of one car to travel to some of the schools in the district (average estimation of 32 US$ per return trip).

Table S2.1. Target girls and coverage for different rounds of vaccination

	Target girls	Girls vaccinated	Coverage
Round 1	2,280	2,365	103.7
Round 2	2,280	2,307	101.2

Round 3	2,280	2,276	99.8

Note: 2,280 10-year-old girls enrolled at school (according to the school-based census) over an estimated total of 2,974 girls of 10 years of age in Manhiça district (according to the National Institute of Statistics).

The Ministry of Education (MoE) incurred no direct expenses but some resources were used during the HPV programme, considered in the analysis as economic costs. Three representatives of the MoE participated in microplanning, social mobilisation, training activities (as recipients of the district level training and as trainers at school level) prior to vaccine delivery. During the first week of the programme kick-off, the representatives moved to the district to supervise and support vaccination activities in coordination with the Manhiça District Directorate of Health. Assuming an average *per diem* of 65 US$, for 3 representatives of the MoE and 7 days of work, a total of 1,365 US$ in resources were allocated to MoE staff by the demonstration program. Additional resources from the MoE included the use of a car during 7 days, implying an average *per diem* of 55 US$ for the driver and around 226 US$ of fuel (160 litres).

Role of Manhiça District Hospital

The key respondents at the Manhiça District Hospital confirmed that, among the 12 health facilities, 11 normally have at least three health professionals: an internist technician, a general nurse and a maternal health technician. One to two health professionals from each facility travelled to every school within the health facility´s catchment area to participate in Information, Education and Communication (IEC) and mobilisation activities and administer vaccines as part of the vaccine delivery activities. The same health professionals were responsible for the waste management at school and later in the health facility.

An average yearly wage of 4,785 US$ for each vaccinator (preventive medicine technician) was considered in the analysis. The team also included a responsible for school health promotion and one teacher to help organise the activities. The teacher brought the students to the area allocated for vaccination, confirmed their names, eligibility and presence, updated the registration book, as well as filled in and managed the vaccination cards.

The role of the Manhiça Health Research Centre (CISM)

Most of CISM activities concerned scientific evaluation of vaccine acceptability, coverage, practicality of reporting adverse events, programme costs and post-vaccine introduction evaluation. These included disease surveillance and monitoring (5,000 US$) and post introduction evaluation (9,000 US$). Additionally, CISM incurred in the evaluation of vaccine delivery strategy (7,000 US$), assessment of feasibility of integrating adolescent health programmes with HPV vaccine (10,000 US$), improvement of the national cervical cancer prevention and control strategy (10,000 US$), coverage survey (26,000 US$) and test HPV vaccine delivery with adolescent health interventions (25,000 US$). All monitoring and evaluation activities amounted to a total of 92,000 US$. These activities were not considered as programme costs as they remained outside the usual EPI vaccination activities. However, CISM supported activities of the Manhiça District Hospital with own funding, such as pre-testing IEC materials in Manhiça (transport, a fieldworker and refreshments for participants) and implementation (transport, personnel for vaccine delivery and social mobilisation), which did not receive funding from the programme and were considered economic costs.

Role of partner Non-Governmental Organisation (NGOs)

Two NGOs participated in the development of the HPV programme in Manhiça district. Village Reach (VR) focused their support at the provincial level, by providing human resources for the vaccine delivery. They spent 161 US$ on the mobilisation and other educational materials for the three districts where the initial programme was launched. They also supported advocacy work by contributing with 50% of *per diems* at provincial level for the personnel in charge of advocating activities.

The Foundation for the Development of the Community (FDC) was the collaborator of the MoH for community mobilisation activities including development and testing of IEC materials, training and mobilising the community to adhere to the campaigns. Their expenses were mainly funded by MoH (through the GAVI grant), but due to delays in reimbursements, some resources were used in advance by FDC to fulfil their IEC and mobilisation objectives. Their activities lasted 15 days in total and included more than 1 week developing and testing the IEC materials, 3 days of activists and radio actors training and 2 days of sensitisation at the community. Sixty people were involved in total and their work focused in Manhiça district, mainly Maluana, Xinavane, Manhiça and 3 de Fevereiro administrative posts. Supervision activities were initially considered but later dismissed due to budget cuts. A total of 7,184.57 US$ were spent in social mobilisation activities by FDC. From this amount, 5,371.61 US$ were considered financial costs transferred from MoH, while 1,812.96 US$ were incurred by FDC and not reimbursed by MoH (economic cost). Additional funds were available for communication materials reprint, airing of radio spots and dissemination of messages for the subsequent doses. These were not transferred to FDC and this activity became responsibility of MoH with the support of CISM.

Table S5. Number of schools and girls censed for vaccination

Administrative Post	School	10-year-old girls (2004)	Censed girls for vaccination
Município	1. EPC Manhiça	84	34
	2. Maragra	143	47
	3. Cambeve	37	34
	4. EP2 - Manhiça	34	18
	5. Maciana	109	61
	6. 7 de Abril	152	143
	7. Ribangua	72	56
	8. Chibututuine	37	33
	9. Timaquene	32	5
	10. Ribjene	15	17
	11. Machavanhane	22	13
	12. Machecane	4	3
	13. Chibucutso	37	32
	14. 24 de Julho	9	6
	15. Muboco	12	10
	16. Malungana	17	13
	17. Malangana	2	4
	18. Nhambi	9	10
	19. Magaba	7	4
	20. Swinhaquene	7	3
	21. Mitilene	18	29
	22. Mulembja	124	84
	23. Eduardo Mondlane	45	48
	24. Marista	18	19
TOTAL Municipio		**1,046**	**726**
Maluana	25. Maluana	45	50
	26. Pateque	42	35
	27. Tavira	25	25
	28. Cuanine	41	33
	29. Munguine	50	41
	30. J. M. Cocolino	19	11
	31. Cantine	40	37
	32. Pembe	16	7
	33. Macandzene	8	4
	34. Barrica	14	7
	35. Serra		25
	36. Bunhe	1	1
	37. Xirindza	11	13

**TOTAL *Maluana***		**312**	**289**
Xinavane	38. EPC 9 de Fevereiro	32	39
	39. EPC Xinavane	32	32
	40. Mepambe	101	65
	41. Aguiar	39	33
	42. Machambutana	30	30
	43. Machambuiana	10	26
	44. Mababe	6	3
	45. Mataquenhane	11	12
	46. Filipe S. Magaia	12	9
	47. Vamagogo	24	12
	48. Santa Rita	21	15
**TOTAL *Xinavane***		**318**	**276**
Calanga	49. EPC Muguejo	3	11
	50. Chécua	7	6
	51. Lagoa Pate	3	3
	52. Chichongue	10	12
	53. Chipuco	6	7
	54. Calanga-Sede	11	6
	55. Pondzene	2	6
	56. Mobana	4	10
	57. Melembene	4	9
	58. Morrumbana	10	7
	59. Halamana	5	1
	60. Chicavele	3	3
	61. Manandze	8	5
	62. Mahila	8	8
	63. Sianine	4	4
	64. Chicuate	7	6
	65. Chingunwine	2	3
	66. Nhacana	3	6
	67. Matlombe	1	3
	68. Bassana	3	7
	69. Mahumana	2	1
	70. Chiau	4	3
TOTAL Calanga		**110**	**127**
Ilha Josina	71. Ilha Josina Machel	63	42
	72. Cutana	22	22
	73. Mampsana	22	22
	74. Marilampfuvo	12	12
	75. Dzonguene	35	25

	76. 1o de Maio	6	5
TOTAL Ilha Josina		**160**	**128**
3 de Fevereiro	77. Palmeira	98	64
	78. Manguendene	99	74
	79. 3 de Fevereiro	51	91
	80. Manchiana	28	23
	81. Chicunguluine	48	47
	82. Milalene	43	49
	83. Taninga	32	10
	84. Chicuachana	69	43
	85. Lhanimane	5	10
	85. Mirrone	3	6
	87. Hunguana	2	2
	88. Pfungurene	12	11
	89. Tchelane	28	20
	90. Melembe		53
	91. Nhavambe	1	1
	92. Condlana	32	16
TOTAL 3 de Fevereiro		**551**	**520**
**TOTAL**		**2,497**	**2,066**

Note: There was a discrepancy between the 10-year-old girls censed by the Ministry of Education (2,497 in 2004) and the estimated by the National Institute of Statistics (2,974 in 2014). The number of primary schools was the same (92).

Table S6: Financial and economic costs of the alternative scenario (US$).

	FINANCIAL COSTS		ECONOMIC COSTS	
**Introduction costs**[1]				
Microplanning and training	2,484	8.80%	6,232	7.10%
Social mobilisation – IEC[2]	3,101	10.99%	3,653	4.16%
Subtotal introduction costs	**5,585**	**19.79%**	**9,885**	**11.27%**
**Recurrent costs**				
Vaccine procurement	10,388	36.81%	52,241	59.56%
Service delivery	2,935	10.40%	13,283	15.14%
Supervision, monitoring & evaluation	7,401	26.22%	7,401	8.44%
Other recurrent costs	1,914	6.78%	1,914	2.18%
Subtotal recurrent costs	**22,638**	**80.21%**	**74,839**	**85.32%**
**Cold-chain supplement**				
Subtotal cold-chain supplement	**0**	**0.00%**	**2,994**	**3.41%**
**Total costs**	**28,223**		**87,718**	

[1]*Introduction costs were annualised in 5 years and only the first year amount was attributable for the 2014 vaccination cycle.*
[2]*IEC: Information, Education and Communication.*
